# A Novel Simulation-Based Multidisciplinary Verbal De-escalation Training

**DOI:** 10.7759/cureus.20849

**Published:** 2021-12-31

**Authors:** Gary Duncan, Megan Schabbing, Brad D Gable

**Affiliations:** 1 Department of Medical Education and Simulation, OhioHealth, Columbus, USA; 2 Department of Psychiatry, OhioHealth, Columbus, USA; 3 Department of Medical Simulation, OhioHealth, Columbus, USA

**Keywords:** communication, standardized patient, staff safety, patient safety, de-escalation, multidisciplinary, simulation

## Abstract

Background

Agitated or aggressive patients pose a high risk of emotional and physical harm to hospital staff. Healthcare associates have the highest rate of workplace violence among studied fields. Learning to effectively de-escalate a patient who is a danger to self or others is key to reducing these incidents. This course was designed to improve education and communication among staff on a hospital surgical floor when verbal de-escalation is needed.

Methods

A ninety-minute simulation-based multidisciplinary curriculum was developed. This included a 30-minute didactic discussion, 10-minute simulation, and 50-minute debrief. Learners included nurses, patient service associates, and protective services officers from a medical/surgical unit. Data were collected using a validated return on investment in learning protocol and the Management of Aggression and Violence Attitude Scale (MAVAS) tool.

Results

Our return on investment in learning showed that more than 97% of learners felt safer in managing agitated patients after participating in the training. The MAVAS tool was used in pre- and post-format and showed a significant trend toward the importance of clear communication and role clarity when de-escalating a patient.

Conclusions

A combination of didactic teaching and simulated experience allowed for greater confidence, communication, and teamwork in de-escalating an agitated patient in a community hospital surgical unit.

## Introduction

Healthcare workers face the risk of verbal and physical assault while at work. According to the Bureau of Labor Statistics, over 70% of workplace assaults occur in healthcare and social service settings, and healthcare workers are more than three times more likely to experience an assault causing missed days of work compared with other fields [1]. In 2019, there were 52 reported deaths in the healthcare industry as a result of workplace violence [2]. Nurses, and especially emergency room (ER) nurses, report an extremely high incidence of workplace violence, with more than 95% of ER nurses and 76% of nurses overall experiencing some form of workplace violence within the past year [3].

Simulation training regarding violent or aggressive patients in the ER and on psychiatric floors has been studied and shown to be beneficial [4]. Commitment to a non-coercive, team-centered approach to agitated patients has been shown to have benefits in safe de-escalation [5]. It is recommended that all types of staff be trained in de-escalation and that a sufficient number of trained staff should be available if a situation arises [6]. A system-level approach to using simulation has been shown to reduce the likelihood of adverse events [7]. Here, we utilized a similar approach to provide education to an in-hospital medical/surgical unit in order to be more prepared for verbal de-escalation of a potentially agitated patient. We identified associates most likely to encounter this situation. These included floor nurses, hospital protective services officers, and patient service assistants (PSAs). Our PSAs perform several tasks in the hospital setting including, sitting with high-risk patients and assisting nursing staff with patient care activities such as meals and turning patients. Our simulation education focused on training for protective services officers, nurses, and PSAs to ensure a multidisciplinary approach to de-escalation. Here, we sought to evaluate a 90-minute simulation-based multidisciplinary education for the de-escalation of agitated patients.

## Materials and methods

Study design and setting

The surgical unit was identified by hospital administration and protective services as having a high number of calls to officers for agitated patients. A group of content experts from psychiatric emergency services, medical education, and simulation worked with unit leadership to determine curricular educational content, goals, and objectives. Since many of the learners had little formal education in de-escalation, we sought to follow the revised Bloom’s taxonomy for providing education [8]. The session would begin with remembering and understanding concepts of de-escalation through a didactic session with facilitated discussion. It would then progress to application and evaluation of the knowledge and skills obtained by the learners through a simulated patient encounter.

Selection of participants 

Learners were identified as a convenience sample of nurses, PSAs, and protective services officers from the surgical unit who were available to attend the education sessions. Sessions were offered on multiple days at variable times to increase participation from staff members from all shifts.

Interventions

A ninety-minute simulation-based education was determined to be the most appropriate method of education by expert consensus. The first 30 minutes entailed didactic education facilitated by a content expert. The objectives for the didactic session were that learners would be able to achieve the following: (1) create a culture of dignity and respect for the patient, (2) show attentiveness to the emotional safety of the patient, (3) develop a framework to empower each staff member to optimally manage a behavioral health emergency, and (4) understand each team member’s role as part of the team when managing a behavioral health emergency. Learners were provided opportunities to ask questions and participate in a facilitated discussion about de-escalation principles. Following the didactic education, learners participated in a simulated patient scenario. In this simulation, the standardized patient portrayed a patient who was becoming agitated after recently transitioning from intravenous to oral pain medications. Learners interacted with the standardized patient using standard hospital protocols and applied new techniques and methods learned during the didactic session. Most simulation sessions took approximately 10 minutes to complete. Following the simulation, a 50-minute debrief was performed and facilitated by expert debriefers. Learners were able to not only reflect on the simulation but also determine how the new de-escalation knowledge and skills could be applied in clinical settings.

Learners participated in the 90-minute simulation-based education in groups of four. Each group consisted of two nurses, one patient service associate (PSA), and one protective services officer. This configuration was determined to be the most realistic number of learners that would need to work together in a clinical setting when de-escalating a patient. A total of 20 education sessions were offered, and some officers and nurses participated twice (although they only completed the post-education survey once).

This project was reviewed by the OhioHealth Institutional Review Board and did not meet criteria for human subjects research, but was considered a quality improvement project.

Measurements

We used two methods to evaluate our educational intervention. The first method entailed a Likert-based scale to measure the impact of the training. These survey questions evaluated (1) self-perceived attitudes and confidence, (2) relevance to clinical practice, (3) likelihood to apply the knowledge, and (4) realism of the simulation. This return on investment in learning (ROL) evaluation is part of our standard learner assessment for all simulation activities and is based on the Phillips return on investment (ROI) methodology [9]. Learners were provided the survey after the 90-minute education. The survey consisted of 12 questions to evaluate the education session. Those questions that evaluated learner reactions consisted of a five-point Likert scale ranging from “strongly disagree” to “strongly agree.” In addition, learners were provided open-ended questions to capture feedback that was not captured elsewhere.

The second method to evaluate this education was the Management of Aggression and Violence Attitude Scale (MAVAS) [10]. This tool has been previously validated for use with staff who commonly interact with patients with psychiatric emergencies. This 30-item survey was conducted in a pre-post format relative to the simulation-based ninety-minute education. Higher scores indicate stronger agreement with each statement using a four-point Likert scale ranging from “strongly agree” = 4 to “strongly disagree” = 1.

Analysis

Survey scores were evaluated using independent sample t-tests to determine if there was an improvement in the Management of Aggression and Violence Attitude Scale (MAVAS) subscales (internal/external, situational/interactional, and management from pre- to post-education training). Statistical analyses used IBM SPSS Statistics version 25.0 (Armonk, NY) based on traditional two-sided tests with the alpha error set at 5%.

## Results

A total of 75 unique learners completed the education (Table 1). Of the 39 employed nurses on the unit, 37 participated in the education, all 20 of the employed PSAs participated, and 17 of the 36 officers who typically provided services at the hospital participated. In order for all PSAs to participate, some officers and nurses completed the education twice, but only completed the MAVAS surveys and the ROL survey the first time they participated. Overwhelmingly, learners agreed or strongly agreed that this training was relevant to their work (97.3%), provided new information or clarified existing education (97.3%), and that they intended to use what they learned (97.3%).

**Table 1:** Number of learners by job description. One learner did not list a job description. PSA: patient service assistant.

Title	Nurse	PSA	Safety officer
Number of learners	37	20	17
Percentage	37/75 (49.3%)	20/75 (26.7%)	17/75 (22.7%)

In addition, learners reported improved confidence with the goals for education (Table 2). Specifically, learners agreed or strongly agreed that they were more confident in the following: providing a culture of dignity and respect for the patient (98.7%), their ability to provide emotional safety for agitated patients (96%), and their ability to safely manage agitated patients (96%).

**Table 2:** Learner responses to return on investment in learning.

Query	Agree/strongly agree	Neutral/disagree
I feel more confident in my ability to provide a culture of respect and dignity for patients	74/75 (98.7%)	1/75 (1.3%)
I feel more confident in my ability to provide emotional safety for agitated patients	72/75 (96%)	3/75 (4%)
I feel more confident in my ability to use a framework to approach agitated patients	69/75 (92%)	6/75 (8%)
I feel more ​confident in my ability to distinguish each team member's role in caring for agitated patients	71/75 (94.7%)	4/75 (5.3%)
I feel more confident in my ability to safely manage agitated patients	72/75 (96%)	3/75 (4%)
This training provided me with the knowledge and skills to provide emotional safety to agitated patients	71/75 (94.7%)	4/75 (5.3%)
This training provided me with the knowledge and skills to use a framework to approach agitated patients	73/75 (97.3%)	2/75 (2.7%)
This training provided me with the knowledge and skills to safely manage agitated patients	73/75 (97.3%)	2/75 (2.7%)

On the 30 item MAVAS evaluation, 11 of the statements showed a significant change from the pre-survey to the post-survey (Table 3). The remaining 19 items did not demonstrate any significant change from pre-survey to post-survey (Table 4).

**Table 3:** MAVAS statements with statistically significant changes pre- and post-simulation. MAVAS: Management of Aggression and Violence Attitude Scale.

Statement	Pre-sim	Post-sim	P-value
Patients are aggressive because of the environment they are in	2.55	2.92	.001
Patients commonly become aggressive because staff do not listen to them	2.76	3.24	.001
It is difficult to prevent patients from becoming violent or aggressive	2.14	2.51	.003
Patients are aggressive because they are ill	2.61	2.94	.003
Poor communication between staff and patients leads to patient aggression	3.14	3.43	.004
Different approaches are used on this ward to manage patient aggression and violence	2.93	3.13	.026
Medication is a valuable approach for treating aggressive and violent behavior	2.94	3.38	< .001
The use of negotiation could be used more effectively when managing aggression and violence	2.97	3.17	.040
Expressions of aggression do not always require staff intervention	2.7	2.41	.018
It is largely situations that contribute toward the expression of aggression by patients	2.85	3.03	.045
Prescription medication should be used more frequently to help patients who are aggressive and violent	2.48	3.19	< .00001

**Table 4:** MAVAS statements without statistically significant changes pre- and post-simulation. MAVAS: Management of Aggression and Violence Attitude Scale.

Statement	Pre-sim	Post-sim	P-value
Other people make patients aggressive or violent	2.72	2.75	.840
Gender mix on the wards is important in the management of aggression	2.68	2.76	.536
Patients from particular cultural groups are more prone to aggression	1.97	2.16	.122
There appear to be types of patients who frequently become aggressive toward staff	2.72	2.75	.840
Cultural misunderstandings between staff and patients can lead to aggression	2.93	3.00	.458
Patients who are aggressive toward staff should try to control their feelings	2.78	2.63	.170
When a patient is violent, seclusion is one of the most effective approaches to use	2.15	2.24	.422
Patients who are violent are often restrained for their own safety	2.73	2.78	.685
The practice of secluding violent patients should be discontinued	2.28	2.33	.584
Aggressive patients will calm down automatically if left alone	2.10	2.02	.436
Restrictive care environments can contribute toward patient aggression and violence	2.99	2.98	.987
Physical restraint is sometimes used more than necessary	2.61	2.73	.326
Alternatives to the use of containment and sedation to manage patient violence could be used more frequently	2.86	2.97	.326
Improved one to one relationships between staff and patients can reduce the incidence of patient aggression and violence	3.28	3.35	.447
Prescribed medication could be handled more effectively on this ward	2.83	2.97	.137
Prescribed medication can in some instances lead to patient aggression and violence	2.97	2.97	.975
Seclusion is sometimes used more than necessary	2.46	2.48	.909
The use of de-escalation is successful in preventing violence	3.12	3.30	.108
If the physical environment were different, patients would be less aggressive	2.47	2.54	.552

Statements that the participants agreed with most strongly after the simulation included the following:

1. Poor communication between staff and patients leads to patient aggression (3.43 on Likert-scale).

2. Medication is a valuable approach for treating aggressive and violent behavior (3.38).

3. Improved one-to-one relationships between staff and patients can reduce the incidence of patient aggression and violence (3.35).

4. The use of de-escalation is successful in preventing violence (3.30).

5. Patients commonly become aggressive because staff do not listen to them (3.24).

6. Prescription medication should be used more frequently to help patients who are aggressive and violent (3.19).

7. The use of negotiation could be used more effectively when managing aggression and violence (3.17).

8. Different approaches are used on this ward to manage patient aggression and violence (3.13).

9. It is largely situations that contribute toward the expression of aggression by patients (3.03).

10. Cultural misunderstandings between staff and patients can lead to aggression (3.00).

All these statements had a weighted score at or above “agree” with a trend toward “strongly agree.” There were no post-simulation statements that had scores <2, which would indicate that no statement had a majority of the respondents that disagreed with the statement. The two most neutral statements, indicating the least agreement among surveyed staff were, “aggressive patients will calm down automatically if left alone (2.02)” and “patients from particular cultural groups are more prone to aggression (2.16).”

## Discussion

Psychiatric emergencies, regardless of the hospital setting, increase the risk of harm to staff, patients, and visitors. Implementing a system of de-escalation that is team-based, easily reproducible, and performed with fully trained staff can help to reduce safety events [4]. Our education emphasized the importance of treating the patient with dignity and respect and understanding the roles of various members of the healthcare team. Including protective services officers in the training was a key aspect of simulation-based education. Security officers in the United States experience workplace injuries at more than twice the rate of the average worker [11]. These crucial staff members are often overlooked when discussing the effects of psychiatric emergencies on healthcare workers. Ensuring thorough, respectful communication with a clear delineation of roles among all associates on the floor leads to better patient outcomes [12,13]. Emphasizing teamwork and patient-centered compassion as key facets of training has shown benefit in tense situations, such as de-escalating a patient, and simulation is well-suited to provide this training in an engaging and effective manner [13-15]. This medical/surgical floor was selected for the training due to a high number of reported incidents in which de-escalation was needed. As a medical-surgical unit, there was limited training in the field of verbal de-escalation that is typically reserved for emergency rooms and psychiatric units. This floor typically has a high number of spinal surgery patients that are being weaned off opiate therapy post-operatively, which has been anecdotally offered as a cause of the increased rate of agitation on the unit.

The multi-disciplinary mixed didactic and simulation curriculum delivered to a medical-surgical unit is novel within the field of de-escalation training. Contracted corporate models for de-escalation training, such as Aegis and Nonviolent Crisis Intervention from the Crisis Prevention Institute (CPI), are solely didactic-based interventions that come at significant cost. This education focuses on early intervention and reducing the need for physical intervention, but without immediate simulation-based training as part of the course [16,17]. Other studies have focused on the simulation of agitation without a structured accompanying didactic component [18]. Our combined approach, utilizing an in-person lecture from a trained emergency services psychiatrist, a structured simulation with standardized patients, and a formal debriefing session, represents a novel approach in this field. Bloom’s taxonomy was utilized for the objectives of the course to identify specific actions that would lead to an improvement in staff confidence and patient care [8]. Objectives measured levels one, two, and five (twice) on Bloom’s levels of learning.

The overall goals for the 90-minute session are outlined in Table 2 above. To achieve these goals, we utilized the 30-minute didactic session to discuss why patients may become agitated, reviewed best practices in verbal de-escalation, and defined the roles of each staff member when attempting to verbally de-escalate a patient. The simulation then focused on utilizing this knowledge and skills. A simulation case was developed using a standardized patient. This patient’s history was taken from recent protective services calls to this floor. The simulated patient had recently undergone a spinal procedure and had been transitioned from IV to oral pain medication. The patient was having severe pain and did not feel that this was being adequately addressed. If the staff were able to recognize why the patient was agitated, employ the empathic skills discussed in the didactic session, and work as a team, then the patient would be able to be de-escalated and the scenario would end. If the staff were unable to perform these functions, then the standardized patient would continue to escalate and the case would be ended at the discretion of the simulation staff and/or subject matter expert. Immediately following the simulation, a fellowship-trained simulation physician facilitated the debriefing session with content expertise provided by the psychiatrist who led the initial didactic session. This debriefing session allowed learners to not only reflect on the knowledge they had gained during the didactic session and applied in the simulation, but also enabled learners to connect these concepts to actual practice and potentially improve how verbal de-escalation is utilized on the medical/surgical unit.

Using the MAVAS both before and after the simulation, we were able to capture the learners' attitudes toward agitated patients. The MAVAS tool has been validated to identify staff attitudes toward patient aggression and the management thereof [10,19]. Our data show that, after participating in the training, staff experienced a significant increase in appreciation for the importance of communication not only between team members but also with the patient. Poor communication and a lack of a streamlined approach to agitation management are likely to result in an increased risk of harm to both patients and staff. The post-simulation responses also showed an increased appreciation for the importance of the environment as a means of potentially driving a patient’s agitation. Positive feelings about increasing negotiation and medication management when attending to agitated patients were also noted by the participants. One participant stated that “using medication soon[er]” and utilizing the “new stalling techniques” in negotiation with the patient was the most helpful skill acquired from the training. One statement on the MAVAS questionnaire that participants felt strongly about both before and after the training was that negotiation was an integral part of de-escalation and could be used more effectively in the future, which underlies the importance of learning about and then putting into action the skills of negotiation in a de-escalation scenario. It is interesting to note that the questions with the largest change in pre- to post-response, as evidenced by p-values <.001, were in the area of using prescription medication in assisting with de-escalation of patients, which indicates a change in participants' perceptions of the effectiveness of that modality in the situation.

A large majority of respondents reported increased confidence in all aspects of the learning objectives for the course. These results included over 98% of learners feeling more confident in providing a culture of respect and dignity for the patient, 96% in providing emotional safety for agitated patients, 92% in their ability to use a specific framework for approaching agitated patients, and 95% in understanding their role in the interdisciplinary team in these situations. Respondents' reactions to the training were overwhelmingly positive, with 97.3% of respondents agreeing or strongly agreeing with the statements: “this training was relevant to my work,” “this training provided me with new information or clarified existing information,” and “I intend to use what I learned from this training.” Multiple participants commented that the debriefing session at the conclusion of the simulation was very helpful because it “allowed the staff to become more familiar” with each other and that “working with and listening to” the other positions was beneficial. One participant commented that “the patient simulation was very authentic and produced feelings similar to a real patient,” and another stated that the scenario was “very realistic! Simulation was life-like and helped display (the) effectiveness of de-escalation.” As stated above, one of the primary learning objectives of the simulation was to create a culture of dignity and respect for the patient. One participant reported that the biggest lesson learned from the course was “having empathy toward patients, putting the behavior and the patient separate.” Suggestions for improvement of the course included comments on increasing the number of scenarios represented and moving the simulation in situ to the unit where the participants worked on a daily basis. One participant also suggested including an informational booklet at the end of the scenario that summarized the information given.

This study focused on learners’ immediate reactions to the simulation training and their attitudes toward managing agitated patients both before and after the education. Our study shows that this simulation-based education for the management of acutely agitated patients is well-liked by learners. Additional studies will focus on patient outcomes such as restraint use and, potentially, the rate of workplace injury on the units that are provided this education. Additional patient-centered outcomes such as time to disposition and time spent in seclusion should also be measured. Staff-specific outcomes such as job satisfaction and staff turnover could also be evaluated.

A limitation of this study is a lack of extended follow-up to assess changes made by staff in their work environment. Several of the MAVAS questions pertain to how patients are managed on the floor, which is better measured after a longer interval than the simulation. A greater change in the areas of action, rather than thinking, may have been present with a follow-up MAVAS questionnaire. This study was conducted on a single medical/surgical unit within a single hospital, so results may not be easily generalizable to other populations. As a single unit study, there was a relatively low total number of participants, and although almost all current unit RNs and PSAs participated, not all safety officers were able to be included in the training. There is also no patient-oriented data to determine whether perceived improvement after completing the training led to improved patient outcomes. In addition, staff-oriented outcomes such as a change in staff assaults or staff reporting feeling more supported in their workplace were not tracked. Additional studies that characterize patient reactions and outcomes, changes in floor policies, or staff-oriented outcomes would be beneficial.

## Conclusions

Combining an expert-led didactic session with a team-based simulation training session improved staff confidence in their ability to de-escalate an agitated patient on a surgical floor. The learning objectives laid out for the course were met, and the method of instruction was shown to be effective.
